# Three kinds of treatment with Homoharringtonine, Hydroxychloroquine or shRNA and their combination against coronavirus PEDV *in vitro*

**DOI:** 10.1186/s12985-020-01342-w

**Published:** 2020-06-03

**Authors:** Cui-Cui Li, Xiao-Jia Wang

**Affiliations:** Key Laboratory of Animal Epidemiology of the Ministry of Agriculture, College of Veterinary Medicine, China Agricultural University, Beijing, 100193 China

**Keywords:** Coronavirus, PEDV, Short hairpin RNA, Heptad repeat, Homoharringtonine, Hydroxychloroquine, Combination treatment

## Abstract

**Background:**

Porcine epidemic diarrhea virus (PEDV) of the family *Coronaviridae* has caused substantial economic losses in the swine husbandry industry. There’s currently no specific drug available for treatment of coronaviruses or PEDV.

**Method:**

In the current study, we use coronavirus PEDV as a model to study antiviral agents. Briefly, a fusion inhibitor tHR2, recombinant lentivirus-delivered shRNAs targeted to conserved M and N sequences, homoharringtonine (HHT), and hydroxychloroquine (HCQ) were surveyed for their antiviral effects.

**Results:**

Treatment with HCQ at 50 μM and HHT at 150 nM reduced virus titer in TCID_50_ by 30 and 3.5 fold respectively, and the combination reduced virus titer in TCID_50_ by 200 fold.

**Conclusion:**

Our report demonstrates that the combination of HHT and HCQ exhibited higher antiviral activity than either HHT or HCQ exhibited. The information may contribute to the development of antiviral strategies effective in controlling PEDV infection.

## Introduction

Porcine epidemic diarrhea virus (PEDV) belongs to the subfamily *Coronavirinae* of the family *Coronaviridae*, which causes epidemic diarrhea in swine, and was first reported in 1971 in the United Kingdom and was soon identified in many European and Asian countries [1]. The incidences of diarrhea occurred in suckling piglets are 80 to 100% on piggeries with an outbreak in China and Southeast Asia [2, 3]. At present, there’s no treatment for PEDVavailable.

The current strategies for controlling viral infectivity focus on identification of agents capable of intervening in the essential steps for viral infection, especially viral entry and replication. The PEDV is an enveloped, positive-sense single-strand RNA virus. The viral genome is approximately 28 kb and includes more than seven open reading frames (ORF) that encode 4 structural proteins, including spike glycoprotein (S), nucleocapsid protein (N), membrane protein (M), and envelope protein (E), 15 non-structural proteins (NSP), and accessory protein ORF3 [1]. N protein is a phosphorylated nucleocapsid protein, and the epitope of N protein can induce the body to produce an effective immune response [4]. M protein plays an essential role in viral assembly [5]. In RNA interference (RNAi), short interfering RNA molecules or short hairpin RNA (shRNA) targeting specific mRNA leads to degradation of mRNA and inhibition of gene expression [6, 7]. RNAi targeting viral genes is an effective way to prevent cells from virus infection, and has been shown to mediate genetic suppression of many human and animal pathogenic coronaviruses [8–10]. Heptad repeat (HR) regions are highly conserved motifs located in spike glycoprotein of enveloped viruses. The viruses such as coronavirus utilize a conserved membrane fusion mechanism, in which highly conserved heptad repeat (HR) regions interact to form six-helix bundle (6-HB) structures, contributing to the fusion of virus and cell membranes. The HIV-1 fusion inhibitor T20, for example, and several modified peptides (e.g., T1249, T2635, T-20 s_138A_), which are derived from the HR region, have been clinically approved to suppress HIV-1 and T-20-resistant variants [11–14]. Coronavirus S glycoprotein shares a mechanism similar to other Class I virus glycoproteins for membrane fusion [15–17]. Our previous work has shown that the modified HR peptide efficiently blocks coronavirus infection [17, 18].

Viruses are able to utilize cellular machineries for viral replication, and a growing understanding of cellular proteins and associated pathways in viral replication has supported the design of new strategies for developing novel antiviral agents. Small molecule chemicals that are able to target cellular machinery act as efficient antiviral agents. The natural compound homoharringtonine (HHT), for instance, is known to inhibit the first cycle of the elongation phase of eukaryotic translation. HHT antagonizes the phosphorylation level of endogenous and exogenous eIF4E (p-eIF4E), which may regulate the selective translation of specific mRNA [19, 20]. In a previous study, we observed that HHT completely inhibited infection by PEDV at a concentration of 500 nM in cell cultures; treatment with HHT at doses of 0.05 mg/kg when after the infection significantly reduced viral load (in the intestine and blood) and relieved severe symptoms in PEDV-infected piglets [20]. One crucial cellular mechanism of autophagy, relies on lysosomes for the clearance and recycling of abnormal proteins or organelles; the deregulation of autophagy is associated with the development of various diseases, including viral infection by PEDV coronavirus [21]. Hydroxychloroquine (HCQ), an autophagy inhibitor chloroquine hydroxy-derivative, acts as an anti-inflammatory agent by attenuating tissue injury through downregulation of inflammatory activation [22]. It also triggers the host defense machinery by inducing ROS- and MAVS-mediated innate immune activation [23]. HCQ shows antiviral activity against a broad spectrum of viruses, including Zika virus and Dengue virus [23, 24].

In the present study, we constructed a truncated HR2 peptide and recombinant lentivirus-delivered shRNAs targeted to conserved M and N sequences, as well as small molecule chemicals HHT and HCQ, to evaluate their antiviral activity. We identified that the combinations of HHT and tHR2 or HCQ more significantly improved the antiviral effects compared to the action of individual components. This paper provides evidence of the potential in vitro antiviral effects against coronaviruses.

## Materials and methods

### Cells, virus and antibodies

Vero cells were cultured in Dulbecco’s modified Eagle’s medium (DMEM) supplemented with 2 mM L-glutamine, nonessential amino acids, sodium pyruvate, 5% or 10% heat-inactivated fetal bovine serum (FBS), 100 U/ml penicillin and 100 μg/ml streptomycin (all reagents are purchased from Gibco Invitrogen). Cells were cultured at 37 °C in a humidified incubator with 5% CO_2_. PEDV strain CV777 stock with 1× 10^7^/ml plaque forming units (PFU) is used in this paper. Anti PEDV-N (1:1000) mouse monoclonal antibody was obtained from Alpha Diagnostic International. Antibodies to actin (1:1000) and goat anti-mouse secondary antibodies conjugated to horseradish peroxidase (HRP, 1:10000) were obtained from Beyotime Biotechnology.

### Design of shRNA plasmids

The shRNA expression vector pGPU6/GFP/Neo was selected to be the parental plasmid. The shRNA sequences were as follows (5′-3′; only the sense strand is shown). shRNA-N: GCAAAGACTGAACCCACTA; shRNA-M: CTGGAATTTCACATGGAAT. The shRNA-MN plasmid carrying two shRNAs was prepared based on the two intermediate plasmids shRNA-N and shRNA-M. Briefly, shRNA-MN DNA sense template was *Bbs* I- (shRNA-N) - hU6 promoter- (shRNA-M) - *Bam*H I. Empty vector pGPU6/GFP/Neo and shRNA-MN sequence were digested with *Bbs* I and *Bam*H I respectively. Then the shRNA-MN sense was ligased to the vector pGPU6/GFP/Neo by T4 DNA ligase. All of these nucleotides were chemically synthesized by Invitrogen.

### Transfection of shRNA plasmids and virus infection

For transfection experiments, 2 × 10^5^ Vero cells were cultured in six-well plates to 50–70% confluence. Cells were transfected with plasmid using TransIntro EL according to the manufacturer’s protocols. Briefly, for each well, 4 μg shRNA plasmid were incubated with 100 μl opti-MEM for 5 min, and 10 μl TransIntro ELwere incubated with 100 μl opti-MEM for 5 min. Subsequently, mixture of 10 μl TransIntro EL and 100 μl opti-MEM was added to the mixture of plasmid and opti-MEM. After incubation for 10–20 min at room temperature (RT), the mixture was added to the well. After incubation at 37 °C for 5 h, the medium containing the transfection mix was replaced with growth medium (DMEM-2%FBS). At 24 h post-transfection (h.p.t), cells were infected with PEDV at multiplicity of infection (MOI) of 0.1. Cultures were then incubated at 37 °C, 5% CO_2_ in a humidified incubator for 1.5 h, at which point the medium was replaced with growth medium. After 48 h of incubation, cells were havested to detect virus titers and the levels of mRNA.

### HHT and HCQ treatment and viral infection

For HCQ, Vero cells were treated with HCQ (25 μM and 50 μM) at the time of PEDV infection (0.1MOI) for 1 h at 37 °C, washed three times with PBS, and overlaid with DMEM-2% FBS containing HCQ with indicated concentrations. At 24 h.p.i., cells were harvested for WB assay and mRNA was extracted for RT-PCR. For HHT, Vero cells were infected with PEDV (0.1MOI) for 1 h at 37 °C, washed three times with PBS, and overlaid with DMEM-2% FBS containing HHT with indicated concentrations. At 48 h.p.i., cells were harvested for WB assay and TCID_50_ assay. For combination of HCQ and HHT experiment, Vero cells were infected with PEDV (0.1MOI) in the absence or presence of HCQ (25 μM and 50 μM) for 1 h at 37 °C, washed three times with PBS, and overlaid with DMEM-2% FBS in the absence or presence of HHT or HCQ with indicated concentrations. At 48 h.p.i., cells were harvested for TCID_50_ assay.

### MTT assay

The MTT assay was conducted according to the manufacturer’s (Beyotime Biotechnology) protocol. Briefly, cells in 96-well plates were treated with shRNA at 37 °C and 5% CO_2_ for 24 h. Then, 10 μl of MTT solution was added to each well and the samples were incubated for 4 h. The medium was then removed and 100 μl of Formazan solution was added to each well. Optical density (OD) values were measured at a test wave length of 570 nm using a Mullikan Ascent Microplate Photometer (Thermo Fisher Scientific, Waltham, MA, USA).

### Preparation of cell lysates and western blotting

The cells in 6-well plate were collected by centrifugation, washed three times with PBS and dissolved in 200 μl lysis buffer (pH 7.5 20 mM Tris-HCl, 150 mM NaCl, 1% Triton X-100, 1 mM EDTA, 2.5 mM Sodium pyrophosphate, 1 mM β-Glycerrophosphate, 1 mM NaVO4, 1 μg/ml Leupeptin) containing protease inhibitor cocktail (Roche) for 30 min on ice. The cell suspension was then fractionated by centrifugation at 6000×*g* for 10 min at 4 °C. Solubilized proteins were harvested, quantified by BCA assay, electrophoresed in denaturing polyacrylamide gels (with 40 μg/well), electro blotted onto a polyvinylidene fluoride (PVDF) membrane, and the antibodies detected the viral protein PVDF. Protein bands were detected with secondary antibody conjugated to horseradish peroxidase (HRP) for 45 min at room temperature, and actin was used as a loading control.

### Quantitative real-time PCR (qRT-PCR)

Cells were harvested, and total RNA was extracted with Trizol (Invitrogen). The RNA was reverse-transcribed into complementary DNA (cDNA). A two-step RT-PCR (SYBR Green I technology, Applied Roche) was performed using SYBR green super mix (Toyobo) according to the manufacturer’s protocol to measure transcription levels for several genes of interest. The primers used were as follows: PEDV-N: 5′- CTG GGT TGC TAA AGA AGG CG − 3′ (forward), 5′- CTG GGG AGC TGT TGA GAG AA − 3′ (reverse). IL-1β: 5′- GAC CTG GAC CTC TGC CCT CTG-3′ (forward), 5′- AGG TAT TTT GTC ATT ACT TTC-3′ (reverse). IL-6: 5′- AAC TCC TTC TCC ACA AGC − 3′ (forward), 5′- TGG ACT GCA GGA ACT CCT − 3′ (reverse). GAPDH: 5′-GAT CAT CAG CAA TGC CTC CT − 3′ (forward), 5′- TGA GTC CTT CCA CGA TAC CA − 3′ (reverse).

Relative fold changes were automatically calculated by the Step One Plus real-time PCR system software (Applied Bio systems), following the 2^-∆∆CT^ method. GAPDH as a control.

### Determination of 50% tissue culture infectious dose (TCID_50_)

Vero cells in 6-well plates were cultured overnight with 80% confluency. Serial dilutions of the PEDV were added to the cells and let them infect of 90 min, and then the culture supernate was replaced with 2% FBS of DMEM and incubated for 48 h. Virus titration was quantified. Ten-fold serial dilutions were prepared for each sample and 100 μl/well of each dilution were added to the cells in 96-well plates in quadruplicates. Wells with cytopathic effect were scored as positive for virus growth and TCID_50_ was determined by the method of Reed and Muench.

### Prediction and construction of HR1 and tHR2

The software packages LearnCoil-VMF (http://nightingale.lcs.mit.edu) and ExPASy-Coils (http://www.ch.embnet.org/software/COILS) were used to study the coiled coils. HR1 and HR2 domains of spike glycoprotein (gS) from the PEDV (GeneBank Accession No. ALS35469.1), derived from amino acids 978 to 1118 and 1263 to 1314 (52Aa), respectively, were predicted. In addition, an optimised Lupas algorithm was used with window widths of 14, 21 and 28 and the MTIDK matrix. The predicted probability of forming coiled coils was 0.9. Optimal focused HR1 and HR2 domains were predicted as follows: NSAIGNITSA FESVKEA ISQTSKGL NTVAH ALTKVQEVVN SQGSALNQLT VQLQHNFQAI, and LTGE IADLEQR SESLRNT TEELRSL INNINNT LVDLEWL (39Aa), respectively. The scramble peptide is LTGE IADVEQR SESVRNT TEEVRSL INNVNNT LVDVEWL, which is also derived from the PEDV HR2 domain, as negative control (NC). All of these peptides were chemically synthesized by China Peptides (http://www.chinapeptides.com/english/index.aspx).

### Gel-filtration analysis

The highly purified HR1 and tHR2 were loaded onto the Superdex G75 column in a solution buffer of 20 mM Tris-HCl, pH 8.0. The peak MW was estimated by comparing the substrate with protein standards running on the same column. The analytical column was calibrated with a series of individual runs of standard molecular mass proteins as markers, including bovine serum albumin (68 kDa), egg white albumin (43 kDa), ribose nucleotides (13.7 kDa), and antimicrobial peptides (5 kDa).

### Circular dichroism (CD) spectra analysis

Peptides were dissolved in PBS (pH 7.4) at a concentration of 30 μM. The wavelength-dependence of molar ellipticity [θ] was monitored at 25 °C using an average of eight scans in a spectropolarimeter (Model J-710, JASCO) with 0.2-cm path length cuvettes from 195 to 245 nm, equipped with a thermoelectric temperature controller. The buffers were filtered in a vacuum pump system with 0.2-μm pore membrane filters. Routine calibration of the machine was performed with d-10 camphor sulphonic acid (60 mg 100 ml^− 1^) according to the eq. [Q] = 100 Q cnl^− 1^, where Q is the ellipticity (mdeg), c is the peptide concentration (mM), n is the number of residues, and l is the path length (cm). Data analyses and acquisition were performed using the spectra manager software provided by the equipment manufacturer. On average, three scans were taken with a scanning rate of 200 nm min^− 1^. The results were expressed as the mean residue ellipticity [Q] (10^− 3^ degree cm^2^ dmol^− 1^). A CD spectra analysis was performed to study the secondary structural changes in single peptides and any combination of two peptides (peptides mixtures) at an equimolar concentration in PBS. In these studies, single peptides were prepared at a concentration of 30 μM, and peptide mixtures at an equimolar concentration in a constant volume (for example, for each peptide mixture, the final concentration of each peptide was 15 μM).

### Immunofluorescence assay

HCQ treatment of PEDV-infected Vero cells (Fig. 3c) growing on glass coverslips in 24-well plates were fixed with 4% paraformaldehyde for 15 min, permeabilized with 0.1% Triton X-100 for 15 min,and blocked with phosphate-buffered saline (PBS) containing 10% horse serum plus 1% bovine serum albumin (BSA) (purchase from Solarbio life sciences) for 2 h. The cells were washed three times with PBS and incubated with diluted primary antibodies overnight at 4 °C. Cells were washed with PBS three times at room temperature (RT), then incubated with fluorochrome-conjugated secondary antibodies in the dark for 1 h at RT. The cells were then rinsed by PBS, mounted on glass slide and examined; images were captured using an microscope Olympus FluoView™ FV1000. The primary antibody to PEDV-N (1:1000) and the goat anti-mouse IgG (H + L) secondary antibody conjugated to Alexa Flour 488 (Invitrogen).

### Statistics

All results were expressed as means and standard deviations (SD). Statistical analyses were performed using Prism 5.01 (GraphPad Software). Significance was determined by one-way analysis of variance (ANOVA) with Dennett’s multiple-comparison test.

## Results

### Dual shRNA-expressing plasmid shRNA-MN was constructed

**Fig. 1:**
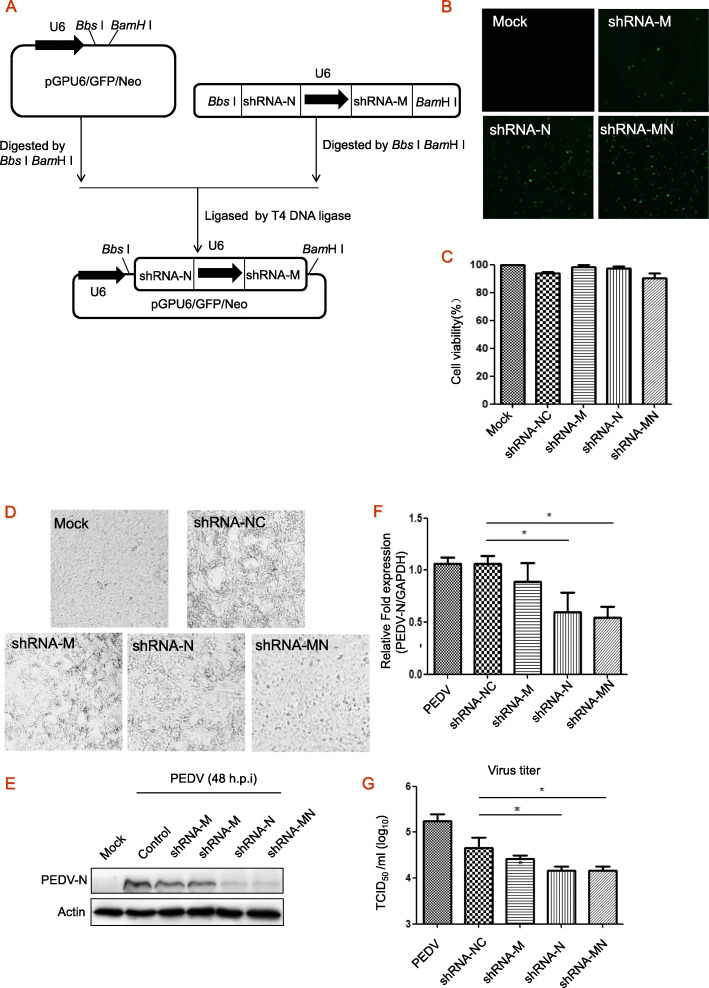
The design of shRNA-expressing plasmids and antiviral activity. **a**, Schematic illustration of construction of the dual shRNA expression plasmid (pGPU6/GFP/Neo-shRNA-MN). **b**, Cells were transfected with shRNA-expressing plasmids for 24 h, transfection efficiency was observed using fluorescence microscope. **c**, The effect on cell viability was assessed by MTT assay. **d**, Cytopathic effects caused by PEDV infection. Vero cells were transfected with shRNA-expressing plasmids for 24 h, and then infected with PEDV at 0.1 MOI. At 24 h post-infection, cytopathic effects were observed using microscope. **e**-**g**, At 48 h post-infection, cells were harvested and immunoblotted with antibody to viral protein PEDV-N, with actin as a control. N/A, the change in abundance of PEDV-N was analyzed by densitometry analysis using Image-Pro Plus Software and normalized to actin. Under the same experimental conditions, total RNA was isolated and PEDV mRNA was quantified by qRT-PCR normalized against GAPDH (**f**), and viral yields in the medium were determined by TCID_50_ (**g**)_._ These experiments were performed two times with three replicates in each experiment. Significance was determined by one-way analysis. Values represent means and SD for triplicates, ^*^p < 0.05

To suppress the replication of PEDV, Vero cells were transfected with shRNA-expressing plasmid targeting the M (shRNA-M) or N (shRNA-N) of PEDV, or shRNA targeting the M and N genes (shRNA-MN). Here, for the dual shRNA expression vector, two shRNAs respectively targeting M and N were incorporated into one vector, pGPU6/GFP/Neo-shRNA-MN, which carries two expression cassettes each containing a U6 promoter. The two corresponding single shRNA-expressing plasmids, pGPU6-shRNA-M and pGPU6-shRNA-N, were constructed and used in parallel (Fig. 1a). Transfection efficiency of shRNA-expressing plasmid shRNA-M was observed by vector GFP fluorescent label (Fig. 1b), and no measurable decrease in cell viability was detected (Fig. 1c).

### shRNA-MN exhibited prophylactic antiviral activity against PEDV infection in Vero cells

Two shRNAs respectively targeting the PEDV M and N genes, and one dual shRNA-expressing plasmid shRNA-MN, were transfected into Vero cells for 24 h before infection to assess the effect of shRNA-expressing plasmids on the replication of PEDV. PEDV-CV777 induced cytopathic effect at 48 h post infection (h.p.i.), while the effect was significantly reduced in shRNA-MN treated cells (Fig. 1d). After 48 h.p.i with PEDV, we observed a significant reduction in viral protein levels (PEDV-N) (Fig. 1e) under treatment with shRNA-N and shRNA-MN, but not with single shRNA-M plasmid. The mRNA levels were significantly inhibited when treated with shRNA-N or shRNA-MN (Fig. 1f). Furthermore, shRNA-MN or shRNA-N reduced 12-fold viral production (Fig. 1g), indicating that the antiviral effect of dual shRNA-expressing plasmids was the same as single shRNA-N.

### HR1 and tHR2 formed a heterotrimer structure

**Fig. 2:**
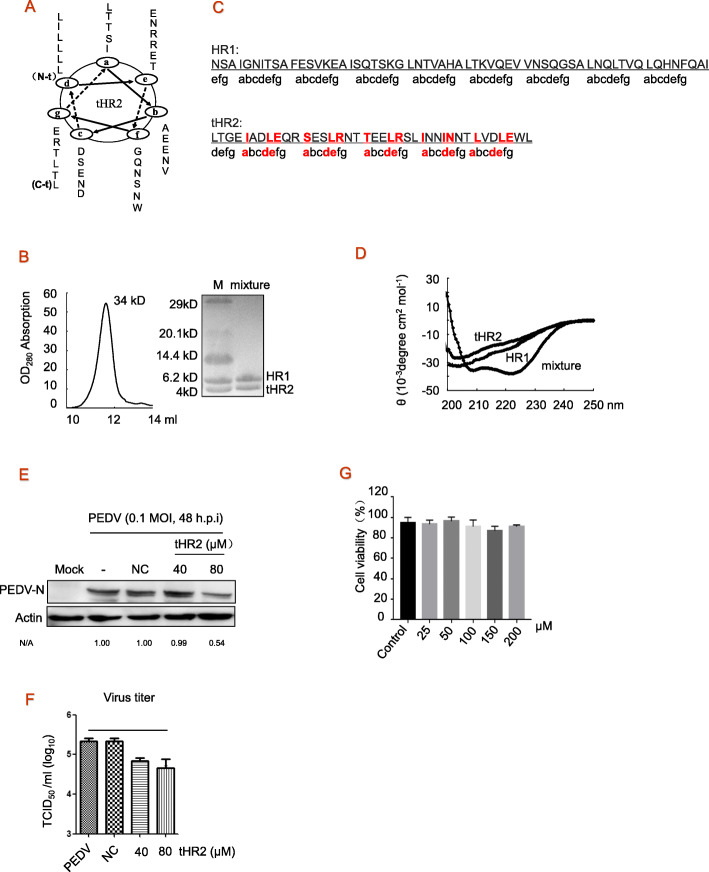
Construction and antiviral activity of soluble peptide tHR2. **a**, The primary structure of each helix consists of heptad repeats (HRs) (**a**–**b**–**c**–**d**–**e**–**f**–**g**) n, in which a, d, and e are interactive sties and **b**, **c**, **f**, and **g** are solvent-accessible sites. Accordingly, the region of residues from 1263 to 1314 (52 residues) is the potential HR2 domain. The truncated HR2 (tHR2) domain contains residues 1278 to 1316 (39 residues). **b**, Gel-filtration analysis of combination of HR1 and tHR2 at the equimolar concentration. **c**, The primary structure of each α-helix consists of a heptad repeat composed of seven residues commonly denoted by (***a****–****b****–****c****–****d****–****e****–****f****–****g***)_n,_ in which **a**, **d**, and **e** are interactive sties. Most important helix-forming residues of site a are highly conserved leucine (L) or isoleucine (I) residues; residues of sites d and e are relatively conserved. **d**, Circular dichroism (CD) spectra analysis. A typical α-helix secondary structure for mixing HR1 and tHR2 was showed. HR1, tHR2 and mixture of both were diluted in PBS for indicated concentrations. The wavelength-dependence of molar ellipticity is monitored at 25 °C as the average of eight scans in a spectropolarimeter. **e** and **f**, Vero cells were treated with scramble (NC) or tHR2 peptides at concentrations of 40 and 80 μM at the time of PEDV infection for 1 h at 37 °C, washed three times with PBS, and overlaid with DMEM-2% FBS containing tHR2. At 48 h post infection, cells were harvested, and viral proteins were detected by western blotting and viral titers were evaluated by TCID_50_ assay. **g**, Effects of tHR2 on cell viability. Cells were treated with various concentrations of tHR2 for 24 h, and cell viability was determined by MTT assay. These experiments were performed two times with three replicates in each experiment. Significance was determined by one-way analysis. Values represent means and SD. * *p* < 0.05 compared to PBS-treated group

The primary structure of each α-helix consists of a heptad repeat composed of seven residues commonly denoted by (*a–b–c–d–e–f–g*)_n_. HR sequences were divided into two characteristic interactive sites (*a, d, e*) and solvent-accessible sites (*b, c, f, g*). The predicted HR2 domain is derived from amino acids 1263 to 1314 (52Aa). Here we truncated the HR2 domain to produce the peptide tHR2 (1278–1316, 39Aa); the relevant helical structures are shown in Fig. 2a.HR1 and tHR2 were produced. The scramble peptide is LTGE IADVEQR SESVRNT TEEVRSL INNVNNT LVDVEWL, which is also derived from the PEDV HR2 domain, as negative control (NC). Determining any direct interaction between HR1 and tHR2, we mixed HR1 and tHR2 peptides, each at 15 μM, and incubated the mixture for 2 h. Using a Superdex G75 column (GE Healthcare), we detected 34 kDa polymers in the chromatographic elutes. The molecular weight (MW) of the HR1 domain from PEDV was approximately 6.5 kDa, and HR2 was approximately 4.3 kDa (Fig. 2b). The 34 kDa polymer may consist of three HR1 and three tHR2 peptides (Fig. 2b). HR1 and scrambled (NC) peptide were not able to bind each other (not shown here). This result indicates that HR1 and tHR2 interacted with each other. Our CD spectroscopic analysis of conformational changes in peptide mixtures revealed a distinguishable value in the HR1 and tHR2 mixture from individual peptides (Fig. 2d), indicating that interaction between HR1 and tHR2 peptides resulted in a different conformation from the homogenous polymers of either peptides. Detecting the effects of tHR2 on cell viability, we treated Vero cells with various concentrations of tHR2 peptide. The results showed that tHR2 peptide did not induce any changes of cell viability (Fig. 2g).

### Peptide tHR2 exhibits antiviral activity against PEDV infection in Vero cells

To test whether the peptides affected virus infection cycle, Vero cells were infected with PEDV at MOI of 0.1 for 48 h in the presence of tHR2 or NC peptide. We found that tHR2 clearly inhibited the expression of viral protein at 80 μM (Fig. 2e), and it also reduced virus titer in TCID_50_ by approximately 4 fold (Fig. 2f). Inhibition by NC peptide was not observed (Fig. 2e, f). Taken together, these results show that exogenous tHR2 showed effective fusion inhibition activity, via competition for the endogenous HR1 region to block six-helix bundle structure formation.

### HCQ regulates autophagy and inflammatory response against PEDV infection

**Fig. 3:**
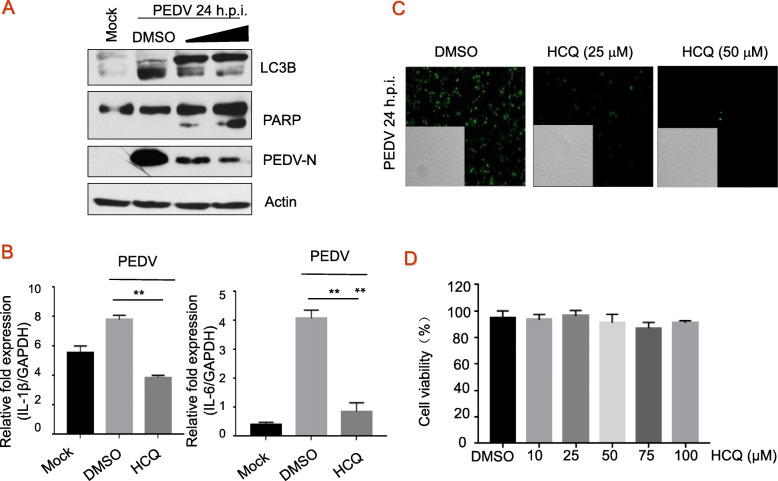
The effect of HCQ on autophagy and inflammatory response. **a**, Vero cells were treated with HCQ at the time of infection for 1 h at 37 °C, washed three times with PBS, and overlaid with DMEM-2% FBS containing HCQ, and LC3B, PARP, and PEDV-N were measured by immunoblotting with specific antibodies, with actin as a control. **b**, Vero cells were mock-infected or infected with PEDV at 0.1 MOI in the presence of the DMSO or HCQ at 50 μM for 24 h, total RNAs were isolated and expression levels of IL-1β (left) and IL-6 (right) mRNAs were quantified by RT-PCR and normalized to that of GAPDH. These experiments were performed two times with three replicates in each experiment. Significance was determined by one-way analysis of variance (ANOVA) with Dennett’s multiple-comparison test. Values represent means and SD, * *p* < 0.05. **c**, Vero cells were infected with PEDV of 0.1 MOI in the presence of HCQ at 25 and 50 μM for 24 h. The infected cells were imaged by fluorescence microscopy using an Olympus IX73 microscope (magnification of 100 ×) equipped with a DP73 camera, and inserted picture is assessed under light microscopy. **d**, Cell viability was assessed by MTT assay according to the manufacturer’s protocols. Each image is representative of three experiments

LC3B is associated with autophagosome development and is used to monitor autophagic activity. Cleavage of PARP is a characteristic of apoptosis. Considering that autophagy is triggered in Vero cells during PEDV infection to promote its replication [21], we explored the effect of autophagy inhibitor HCQ on PEDV infection. As shown in Fig. 3a, HCQ reduced viral infection by blocking autophagy activation induced by PEDV in a dose-dependent manner; it seems that HCQ improved apoptosis via depressing autophagy. RT-qPCR analysis of cells treated with HCQ (50 μM) showed decreased expression of pro-inflammatory cytokines IL-1β and IL-6 (Fig. 3b). Vero cells were infected with PEDV of 0.1 MOI in the presence of HCQ at 25 and 50 μM for 24 h. An indirect immunofluorescence assay was further carried out to verify the antiviral activities of HCQ, and result indicated that HCQ at 25 μM reduced about 90% virions and almost completely inhibited PEDV replication at 50 μM compared to DMSO (Fig. 3c). These results indicate that HCQ reduces PEDV-induced autophagy and inflammatory response. Effects of HCQ on the viability of Vero cells were determined by a MTT assay. Changes in cell viability was not detectable (Fig. 3d).

### Antiviral combination against PEDV infection

**Fig. 4:**
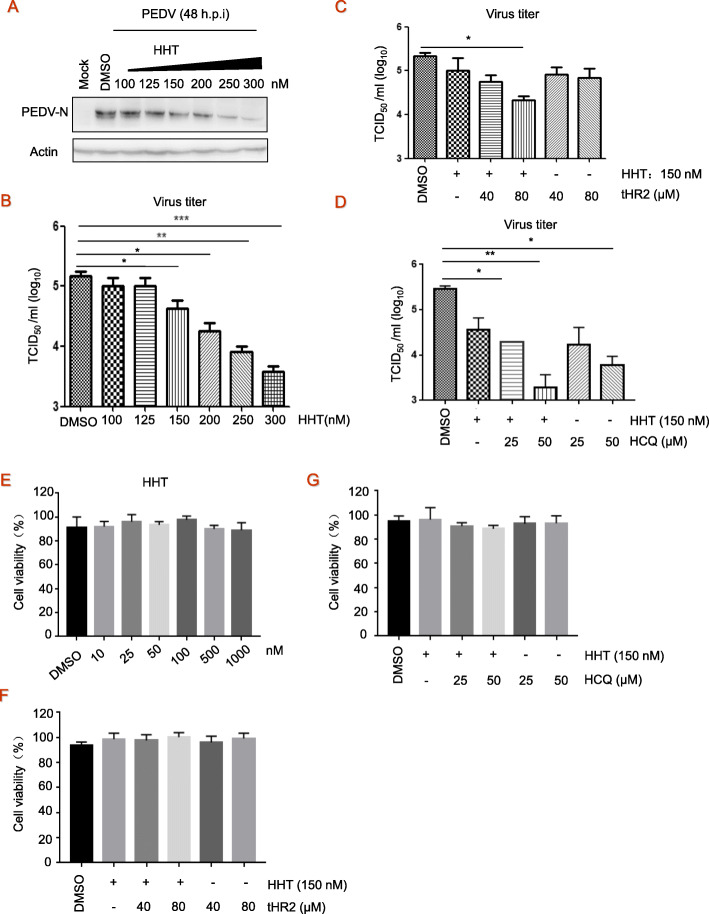
Antiviral activity of combination treatment. **a**-**b**, Vero cells seeded at 2.5 × 10^6^ cells per well in six-well plates were infected with PEDV at 0.1 MOI in the presence of the HHT at different concentrations for 48 h, electrophoretically separated proteins were analyzed by immunoblotting with antibodies to viral protein PEDV-N and actin (**a**). Viral yields in the medium were determined by TCID_50_ (**b**). These experiments were performed two times with three replicates in each experiment. Values represent means and SD. * *p* < 0.05; ** *p* < 0.01; *** *p* < 0.001 compared to PBS-treated group. **c**-**d**, Vero cells seeded at 2.5 × 10^6^ cells per well in six-well, cells were then infected with PEDV at 0.1 MOI in the presence of peptide tHR2 and HHT (**c**) or HCQ (**d**). At 48 h post-infection, virus titer in TCID_50_ was determined. **e**-**g**, Cytotoxicity of HHT and combination treatment was detected by MTT assay. These experiments were performed two times with three replicates in each experiment. Values represent means and SD for triplicates. ^*^p < 0.05, ^**^p < 0.01 for *one-way* ANOVA

Whether a combination of these agents will be optimal to control various viruses remains to be determined. Here, a gradual reduction in PEDV-N protein levels was observed when cells were treated with increasing doses of HHT (Fig. 4a). That is, HHT at 150 nM reduced PEDV virus titer in TCID_50_ by 3.5 fold, and at 300 nM reduced virus titer by approximately 40 fold (Fig. 4b). To further optimize the effects of the antivirals indicated above, the combination of tHR2 at 40 μM and 80 μM with HHT at 150 nM was designed. Treatment of Combination of HHT and tHR2 when during and after the infection resulted in an additive effect on suppression (10-fold of TCID_50_) of viral production/infection at 48 h.p.i (Fig. 4c). For combination of HCQ and HHT experiment, Vero cells were treated with HCQ (50 μM) during infection for 1 h at 37 °C, washed three times with PBS, and overlaid with DMEM-2% FBS in the absence or presence of HHT or HCQ with indicated concentrations. At 48 h.p.i., cells were harvested for TCID_50_ assay. Notably, HCQ at 50 μM reduced PEDV virus titer in TCID_50_ by 30 fold, and the combination with HHT at 150 nM has addictive effects on infection and reduces TCID_50_ by 200 fold. We studied if HHT or combined tHR2 with HCQ was able to modulate Vero cell viability. The results did not reveal any effects all HHT and combinations with tHR2 and HCQ on cell viability (Fig. 4e to g). These findings demonstrate that HCQ effectively inhibits PEDV infection, and the combination treatment at low concentrations produced significantly improved antiviral effect as compared to the separate components.

## Discussion

In the current study, we found that the combination of HHT (150 nM) and HCQ at low concentrations (50 μM) reduced TCID_50_ by 200 fold (Fig. 4). In our previous study, we found that HHT exerted broad-spectrum antiviral action and completely inhibited infection by PEDV at a concentration of 500 nM in Vero cells [20]. Treatment with HHT at doses of 0.05 mg/kg when after the infection significantly reduced viral load (in the intestine and blood) and relieved severe symptoms in PEDV-infected piglets, but treatment with 0.2 mg/kg of HHT led to severe side effects, fatal to about 50% of the piglets [20]. The inhibitory effect of HCQ is associated with host defense machinery and immune regulation; HCQ at high concentration (> 20 μM) also exhibits cell toxicity [21–24]. The study of preparations therefore provides new ideas, but the damage of host cells resulting from increasing doses is a problem that cannot be ignored.

Recent research has shown that combination therapy may be more effective than monotherapy. IFN-β in combination with ribavirin act highly synergistically on production of infectious virus titres and may be highly effective not only as prophylactic agent but also for the treatment of already infected SARS patients [25]. A combination of RNAi and a virus receptor trap exerts additive antiviral activity in coxsackievirus B3-induced myocarditis in mice [26]. Likewise, anti-TNF antibody administered with Sunitinib significantly reduces vascular leakage and synergizes to provide superior protection from lethal DENV infection compared with either agent alone in vivo [27]. Combination therapies targeting viruses at different stages of their infection cycle are a common strategy to improve the efficiency of the antiviral treatment, and the combination of multi-target preparations is expected to be less cytotoxic [28].

The RNAi-based strategy has different mechanisms of action than antibodies, small molecules, and other protein drugs. It is worth mentioning that the RNAi drug Onpattro (patisiran) was the first FDA-approved for adult patients with peripheral neuropathy (polyneuropathy) caused by hereditary transthyretin amyloidosis (hATTR) [29]. Methods to express multiple shRNAs from a single vector have been explored, including expression from multiple cassettes using the same promoter [30], or from a single vector containing multiple promoters [31]. The inhibition of viruses by means of RNAi is an approach to antivirals that has gained importance in recent years [10, 32–35], but the use of multiple shRNAs in the elimination of viral infection has been less successful. In this paper, we established the dual shRNA expression vector, targeting M and N genes, which carries two U6 promoters. We have established a concept for developing transfection vectors that may have wide applications in gene antiviral strategies, including the delivery of siRNA to combat viral infection. This approach has the potential to be utilized for the prevention and control of coronavirus PEDV [36]. However, our study demonstrates that the antiviral effect of dual shRNA-expressing plasmids was the same as single shRNA-N. This result may be related with low transcription efficiency or low antiviral activity of shRNA-M in dual shRNA-expressing plasmid. Therefore, we think double shRNA needs to be improved for high transcription efficiency and antiviral activity. In the future, the antiviral effects of shRNA will be evaluated in vivo. It is proposed that porcine U6 promoter-based shRNA vector might have more transcriptional activity than human U6 promoter in piglet.

The membrane fusion process has been considered as a significant antiviral target and has received wide attention; the recombinant HR2 region of fusion glycoprotein is the ideal fusion inhibitor for class I enveloped viruses [11]. Based on a LearnCoil-VMF prediction result, amino acid L1252 to L1286 (35Aa) from the HR2 domain of S glycoprotein was selected to design a fusion inhibitor that efficiently blocks MERS-CoV entry [16, 17]. The predicted HR2 domain (65Aa) from S glycoprotein of PEDV, however, is longer than that in MERS-CoV and other coronaviruses [16–18], and synthetic HR2 peptide at the concentration of 40 μM statistically inhibits PEDV infection [37]. Reducing the length of the active peptide may be an important consideration to reduce the cost of peptide agents. Here we truncated the tHR2 domain to produce the peptide tHR2 (39Aa), with the aim of identifying a minimal domain capable of disrupting the formation of the six-helix bundle required for viral cell entry. Our results suggest that tHR2, but not scrambled peptide, has the ability to inhibit PEDV infection (Fig. 3e, f). Furthermore, the antiviral effect of tHR2 is not as strong as expected when compared to recombinant HR2 peptides (as shown in [17]), perhaps due to the short length or disrupted core structure. It is noteworthy that by 2013 the FDA had already approved the listing of more than 60 peptide drugs [38]. Peptides will be widely used in medicine and biotechnology in the near further.

In this study, we explored how to maximize the antiviral effect of HHT while reducing host cell toxicity. We found that tHR2 peptide designed based on PEDV S genes effectively inhibits the membrane fusion of PEDV when added during and after the infection, via competition for the endogenous HR1 region to block six-helix bundle structure formation and does not affect the cell viability. The combination treatment with HHT when added after the infection and tHR2 when added during and after the infection results in additive protective effects by inhibiting membrane fusion and viral replication. We also found that the combination of HHT when added after the infection and pre-transfected shRNA-MN did not exhibit improved effects (not shown here), perhaps because they have the same target in the viral replication process. Short hairpin RNA (shRNA) targeting specific mRNA leads to degradation of mRNA and inhibition of gene expression and HHT may regulate the selective translation of specific mRNA to inhibit viral gene expreesion. We propose that they may have same target during process of gene express. The combination treatment with HHT when added after the infection and HCQ when added during and after the infection results in additive protective effects. The combination of a low dose (150 nM) of HHT and/or HCQ as host targeting antivirals (HTA) with tHR2 as direct-acting antiviral (DAA) has the potential to improve safety and increase the antiviral effect. These results provide a reference for the development of clinical drugs against coronavirus infection in the future.

## Conclusion

This paper provides information to contribute to the development of effective antiviral strategies against PEDV coronavirus. Our proof-of-concept study identified the combination of HHT with HCQ at low concentration as having an significant high antiviral effectiveness. The combination exerts additive effects and is significantly more effective than either of the treatments separately in inhibiting PEDV infection in vitro.

## Data Availability

All data generated or analyzed during this study are included in this published article.

## Abbreviations

h.p.i.: Hour post infection
HCQ: Hydroxychloroquine
HHT: Homoharringtonine
HR: Conserved heptad repeat regions
PEDV: Porcine epidemic diarrhea virus
PFU: Plaque forming units
RNAi: RNA interference
RT: Room temperature
SD: standard deviations
ShRNA: Short hairpin RNA
